# Intrauterine inflammation exacerbates maladaptive remodeling of the immature myocardium after preterm birth in lambs

**DOI:** 10.1038/s41390-022-01955-7

**Published:** 2022-03-11

**Authors:** Amanda Vrselja, J. Jane Pillow, Jonathan G. Bensley, Stacey J. Ellery, Siavash Ahmadi-Noorbakhsh, Timothy J. Moss, M. Jane Black

**Affiliations:** 1 Department of Anatomy and Developmental Biology, Monash Biomedicine Discovery Institute, Monash University, Clayton, VIC Australia; 2 School of Human Sciences, University of Western Australia, Perth, WA Australia; 3 The Ritchie Centre, Hudson Institute of Medical Research, Department of Obstetrics and Gynaecology, Monash University, Clayton, VIC Australia

## Abstract

**Background:**

Antenatal conditions that are linked with preterm birth, such as intrauterine inflammation, can influence fetal cardiac development thereby rendering the heart more vulnerable to the effects of prematurity. We aimed to investigate the effect of intrauterine inflammation, consequent to lipopolysaccharide exposure, on postnatal cardiac growth and maturation in preterm lambs.

**Methods:**

Preterm lambs (~129 days gestational age) exposed antenatally to lipopolysaccharide or saline were managed according to contemporary neonatal care and studied at postnatal day 7. Age-matched fetal controls were studied at ~136 days gestational age. Cardiac tissue was sampled for molecular analyses and assessment of cardiac structure and cardiomyocyte maturation.

**Results:**

Lambs delivered preterm showed distinct ventricular differences in cardiomyocyte growth and maturation trajectories as well as remodeling of the left ventricular myocardium compared to fetal controls. Antenatal exposure to lipopolysaccharide resulted in further collagen deposition in the left ventricle and a greater presence of immune cells in the preterm heart.

**Conclusions:**

Adverse impacts of preterm birth on cardiac structure and cardiomyocyte growth kinetics within the first week of postnatal life are exacerbated by intrauterine inflammation. The maladaptive remodeling of the cardiac structure and perturbed cardiomyocyte growth likely contribute to the increased vulnerability to cardiac dysfunction following preterm birth.

**Impact:**

- Preterm birth induces maladaptive cardiac remodeling and adversely impacts cardiomyocyte growth kinetics within the first week of life in sheep.
- These effects of prematurity on the heart are exacerbated when preterm birth is preceded by exposure to intrauterine inflammation, a common antecedent of preterm birth.
- Inflammatory injury to the fetal heart coupled with preterm birth consequently alters neonatal cardiac growth and maturation and thus, may potentially influence long-term cardiac function and health.

## Introduction

Globally, 1 in 10 infants is born preterm (<37 weeks of gestation).^1^ Chorioamnionitis is a major risk factor for preterm birth.^2,3^ Chorioamnionitis arises from a polymicrobial infection and causes inflammation of the fetal membranes (the chorion and amnion) triggering a maternal and fetal inflammatory response.^2,3^ Consequently, chorioamnionitis may influence the structure and function of fetal and neonatal organ systems.^4,5^ Observations in preterm infants suggest that intrauterine inflammation adversely impacts cardiovascular function. For example, preterm infants exposed to chorioamnionitis exhibit elevated heart rates^6^ and an increased risk of hypotension^7^ during the first 12–24 h of life.

The intrauterine inflammation associated with chorioamnionitis is mimicked in experimental models by an intra-amniotic administration of endotoxin, such as lipopolysaccharide (LPS; derived from the outer wall of gram-negative bacteria).^8–10^ The fetal immune system recognizes LPS as a pathogen-associated molecular patterns molecule and elicits an inflammatory response.^10^ A potential primary target for LPS-induced inflammation is the heart.^11,12^ Fetal sheep exposed to LPS develop tachycardia and hypotension,^13,14^ which are associated with impaired myocardial function.^9,13^ In addition to disturbed cardiac function, fetal sheep exposed to LPS have increased collagen deposition in the right ventricle and accelerated cardiomyocyte maturation, hallmarked by an increase in the size and proportion of binucleated cardiomyocytes.^8^ While structural changes in the developing fetal myocardium occur in response to intrauterine exposure to LPS,^8^ the postnatal effects of in utero exposure to LPS are unknown.

Independent of chorioamnionitis, preterm birth imposes increased functional demands on the infant’s immature cardiovascular system, due to the premature hemodynamic transition at birth.^15^ Unlike the hearts of term-born infants, preterm infants are born during an important period of myocardial growth and maturation, and hence, premature delivery may adversely impact final cardiomyocyte endowment.^16^ A unique cardiac geometry in preterm-born individuals is observed from early neonatal life with distinct differences in ventricular mass, volume, and shape^17,18^ that persists into adulthood with changes in function and evidence of myocardial fibrosis.^19–21^ Preclinical animal studies also highlight maladaptive remodeling of the myocardium in response to preterm birth.^22–24^

As intrauterine inflammation often precedes preterm birth, it is paramount to understand the consequences of both of these adverse events on the newborn heart. We hypothesized that cardiac structure and maturation are impacted adversely by prematurity and that these adverse effects are exacerbated by prior exposure to intrauterine inflammation. The aim of this preclinical sheep study was to determine the effect of preterm birth with or without intrauterine LPS exposure (used to evoke a fetal inflammatory response) on cardiac structure and expression of cardiac target genes, as well as cardiomyocyte maturation and endowment in the first week of life.

## Methods

### Ethical approval

The animal experiments were approved by the Animal Ethics Committee at the University of Western Australia (RA 3/100/1301) and were performed in accordance with the Australian Code for the care and use of animals for scientific purposes.

### Animal groups

Date-mated pregnant merino ewes received an intramuscular injection of medroxyprogesterone (150 mg; Depo-Provera, Pfizer, Australia) seven days before cesarean delivery. Ewes were randomized to receive either an intra-amniotic injection of LPS (preterm LPS, *n* = 9; 4 mg, *Escherichia coli*, O55:B5, Sigma-Aldrich, Saint Louis, Missouri), or saline (Preterm SAL, *n* = 9) as an experimental control, 48 h before preterm delivery. Maternal intramuscular betamethasone (5.7 mg) was given at 6 and 24 h post-intra-amniotic injection. Lambs were delivered preterm at ~129 days of gestational age (term is ~150 days of gestational age). Postnatal care of lambs followed contemporary clinical management including surfactant (poractant alfa: Chiesi Farmaceutici S.p.A., Parma, Italy), targeted supplemental oxygen administration, and a progressive de-escalation of respiratory support and extubation to non-invasive support as soon as possible following delivery. Lambs were humanely killed at seven days of postnatal age (~136 days of postconceptional age), with a lethal intravenous injection of pentobarbitone (150 mg/kg). Gestational age-matched naive fetal controls (fetal control, *n* = 7) were humanely killed at 136 days of gestational age. At necropsy, hearts were excised and weighed. Ventricular tissue was sampled (1 cm^3^) from the right ventricular (RV) free wall and from the apex of the left ventricle (LV) and snap-frozen for molecular analyses. Hearts were perfusion fixed via the aorta as described previously.^22,25^

### Cardiac sampling and morphological assessments

The atria and great vessels were removed from the fixed hearts and the ventricles and adjoining septum cut transversely into 10 mm thick slices. The second slice from the base of the heart was imaged for measurement of the LV, RV, and interventricular septal (IVS) wall thickness (Image-Pro Plus Version 6.0, Media Cybernetics, Rockville, Maryland).^22,25^ The RV and left ventricle plus septum (LV + S) were separated for independent analyses and were sampled randomly using a smooth fractionator approach.^22,25,26^ Sampled tissues were processed and embedded in glycolmethacrylate or paraffin for comprehensive analysis of the ventricular structure and cardiomyocyte growth, described briefly below. A detailed description of the following methods is provided in the supplementary material online.

### Assessment of myocardial collagen

Paraffin-embedded ventricular samples were sectioned at 5 µm and pre-treated with Bouin’s fixative and stained with picrosirius red. Sections were scanned at ×20 magnification and exported into Aperio Imagescope (Version 12.3.3, Leica Biosystems, Vista, California). Approximately 50–75 fields of view (339,690 µm^2^) were analyzed for quantification of interstitial collagen content using an optimized Positive Pixel Count (v.9) algorithm in Aperio Imagescope.^27^

### Quantification of immune cell infiltration

Paraffin-embedded sections immunohistochemically labeled to identify CD45 (common leukocyte antigen) were scanned at ×40 magnification and exported into Aperio Imagescope.^28^ The average number of CD45^+^ cells per unit area was determined in ~50 random fields of view (13,045.75 µm^2^) per ventricle.

### Quantification of cellular proliferation

Paraffin-embedded ventricular sections were labeled immunohistochemically for the proliferative cell marker Ki67. The slides were scanned at ×40 magnification and exported into Aperio Imagescope where each slide was manually assessed to exclude proliferating non-cardiomyocyte cells with inclusion and exclusion zones drawn. The modified images were analyzed and the proportion of Ki67-positive cardiomyocyte nuclei within the myocardium was determined using an optimized nuclear count algorithm (Aperio Imagescope).^27^

### Measurement of cardiomyocyte cross-sectional area and nuclearity

Cardiomyocyte cross-sectional area and nuclearity were quantified in paraffin sections stained with the nuclear stain DAPI (4′,6-diamidino-2-phenylindole; Invitrogen, Carlsbad, California) and Wheat Germ Agglutinin (WGA) conjugated to Alexa-Fluor 488 (Invitrogen), which stains the cell membranes.^29,30^ Cardiomyocytes were measured in cross-section (an index of cell size) for evidence of cellular hypertrophy.^29^ The cross-sectional area of more than 300 cardiomyocytes with a centrally located nucleus was measured per ventricle.^29^ Cardiomyocyte nuclearity is a useful marker of cardiomyocyte maturation, with binucleation usually indicative of cardiomyocyte maturation and differentiation in sheep cardiomyocytes.^31^ Images in the longitudinal plane were analyzed through the *Z*-stack to visualize and quantify nuclearity in more than 250 cardiomyocytes for each animal.^22^

### Stereological estimation of cardiomyocyte endowment

The total number of cardiomyocyte nuclei per ventricle was estimated in glycolmethacrylate-embedded sections using an optical disector/fractionator technique,^32^ as described previously.^22,33^ Total cardiomyocyte number was then determined by adjusting for cardiomyocyte nuclearity.

### RNA extraction

The purification of total RNA from LV and RV tissue was conducted using the PureLink™ RNA Mini Kit (Invitrogen, Life Technologies, Carlsbad, California) with TRIzol® Reagent (Ambion, Life Technologies, Carlsbad, California), according to manufacturer’s instructions. Total RNA concentration was quality assessed by spectrophotometry (NanoPhotometer® N50; Implen, Munchen, Germany) and stored at −80 °C.

### cDNA synthesis

Complimentary DNA (cDNA) was synthesized from total RNA using the SuperScript® III First-Strand Synthesis System (Invitrogen, Life Technologies). A negative control, -Reverse Transcriptase (-RT) control, was prepared with all components for cDNA synthesis except for the SuperScript® III Reverse Transcriptase. Samples were incubated in the thermal cycler (Veriti™ 96-Well Thermal Cycler; Applied Biosystems, Life Technologies) and stored at −20 °C.

### Gene expression

All samples were quality checked by SYBR™ chemistry real-time polymerase chain reaction (PCR) for gDNA contamination using the 7900HT Fast Real-Time PCR System (Applied Biosystems, Life Technologies). The TaqMan® Gene Expression Assay (Applied Biosystems, Life Technologies) was used to quantify gene expression. The genes of interest (listed in Table 1) examined have functions relating to angiogenesis, calcium handling, collagen synthesis, extracellular matrix modulation, inflammation and cardiac hypertrophy, metabolism and remodeling. Real-time PCR was performed using the Biomark™ HD (Fluidigm, South San Francisco) dynamic integrated fluidic circuit; representative group samples were run in duplicate. The data were analyzed with Fluidigm Real-Time PCR analysis software (version 4.1.1; Fluidigm) to output sample Ct values.

**Table 1:** List of genes examined in the hearts of fetal and preterm sheep.

Symbol	Name	Summary	TaqMan® probe ID
*ATP2A2*	ATPase sarcoplasmic/endoplasmic reticulum Ca^2+^ transporting 2	Calcium handling	Oa01201433_m1
*COL1A1*	Collagen type I α 1 chain	Collagen synthesis	Oa01463861_gH
*COL3A1*	Collagen type III α 1 chain	Collagen synthesis	Oa04910910_m1
*GATA4*	GATA binding protein 4	Cardiac hypertrophy	Oa04298610_m1
*IGF-1*	Insulin-like growth factor 1	Cardiac hypertrophy	Oa04657098_m1
*IL-1b*	Interleukin 1β	Inflammation; cardiac remodeling	Oa04656322_m1
*IL-18*	Interleukin 18	Inflammation; cardiac remodeling	Oa04658606_m1
*MMP9*	Matrix metallopeptidase 9	Cardiac remodeling	Oa03215996_g1
*MYH7*	Myosin heavy chain 7	Cardiac hypertrophy	Oa04876075_g1
*NPPA*	Natriuretic peptide A	Cardiac hypertrophy	Oa04657625_g1
*PPARA*	Peroxisome proliferator-activated receptor α	Cardiac metabolism	Oa04912809_m1
*PPARGC1A*	Peroxisome proliferator-activated receptor γ coactivator 1 α	Cardiac metabolism	Oa01208835_m1
*RYR2*	Ryanodine receptor 2	Calcium handling	Oa04679731_m1
*SLC8A1*	Solute carrier family 8 member A1	Calcium handling	Oa04866710_m1
*TIMP2*	TIMP metallopeptidase inhibitor 2	Extracellular matrix modulator	Oa04655716_m1
*TGFβ1*	Transforming growth factor β1	Inflammation; cardiac remodeling	Oa04259484_m1
*TLR4*	Toll-like receptor 4	Inflammation; cardiac remodeling	Oa04656419_m1
*TNF*	Tumor necrosis factor	Inflammation; cardiac remodeling	Oa04655425_g1
*VEGFA*	Vascular endothelial growth factor A	Angiogenesis	Oa04653812_m1

### Gene expression analysis

Ct values were analyzed using the qBase+ software (version 3.1; Biogazelle, Gent, Belgium).^34^ The reference gene *YWHAZ* was selected based on the qBase+ geNorm algorithm. The expression of all genes was provided as the calibrated normalized relative quantity (CNRQ; normalized to *YWHAZ* and expressed relative to the fetal control group), where the fetal control values were normalized to 1.00.

### Sample size and statistical analyses

The number of lambs was determined using a power analysis based on the previously reported variation from our laboratory^22,25^ associated with the techniques of the primary morphological and stereological endpoints, which were the main outcomes of this study.

Statistical analyses were performed using GraphPad Prism (Version 7.0b, GraphPad Software, California). All data were tested for normality using a Shapiro–Wilks test and analyzed using a one-way ANOVA followed by a Bonferroni post hoc test. Statistical analyses for gene expression were performed in qBase+ using a one-way ANOVA followed by a Tukey–Kramer post hoc test. Data are expressed as the mean ± SD and a *p* value <0.05 was considered significant.

## Results

### Birthweights and body and heart weights at postnatal day seven

All preterm lambs were lighter at birth (preterm SAL vs fetal control, *p* < 0.0001; preterm LPS vs fetal control, *p* = 0.002) and at necropsy (postnatal day 7; *p* < 0.0001) than age-matched control fetuses; refer to Table 2. Likewise, hearts were lighter at necropsy in preterm lambs compared to age-matched control fetuses (preterm SAL vs fetal control, *p* = 0.013; preterm LPS vs fetal control, *p* = 0.05), but no differences when adjusted for body weight.

**Table 2:** Overview of fetal and preterm lambs at delivery and necropsy.

	Experimental groups		
	Fetal control	Preterm SAL	Preterm LPS
Gestational age at delivery (days)	136.6 ± 1.0	129.7 ± 1.4	128.9 ± 1.0
Postconceptional age at necropsy (days)	136.6 ± 1.0	137.9 ± 1.9	135.4 ± 1.3
*N* (male)	7 (4)	9 (4)	9 (4)
Body weight at delivery (kg)	4.05 ± 0.45	2.90 ± 0.41*	3.22 ± 0.37*
Body weight at necropsy (kg)	4.05 ± 0.45	2.79 ± 0.39*	2.97 ± 0.24*
Heart weight (g)	29.4 ± 3.7	23.7 ± 3.1*	24.8 ± 3.8
Heart weight: body weight at necropsy (g/kg)	7.32 ± 1.18	8.64 ± 1.61	8.36 ± 1.05
LV wall thickness (mm)	6.6 ± 0.8	7.3 ± 0.6	7.5 ± 0.8
IVS wall thickness (mm)	7.6 ± 0.9	6.1 ± 0.9*	6.2 ± 0.6*
RV wall thickness (mm)	5.0 ± 0.5	3.8 ± 0.4*	4.5 ± 0.4^†^

Statistical analyses were performed using a one-way ANOVA followed by a Bonferroni post hoc test. Data are presented as mean ± SD. Significance accepted at *p* *<* 0.05: *significant difference compared to fetal control, ^†^significant difference compared to preterm SAL.

### Cardiac morphometry

Data on gross cardiac morphometry are presented in Table 2. At necropsy, the wall thickness of the LV free wall was similar between the groups, whereas the IVS was thinner in the preterm lambs compared to fetal controls (preterm SAL vs fetal control, *p* = 0.005; preterm LPS vs fetal control, *p* = 0.007). Preterm SAL lambs had a thinner RV wall compared to preterm lambs exposed to antenatal inflammation (*p* = 0.017) and fetal controls (*p* < 0.0001).

### Myocardial collagen content

**Fig. 1:**
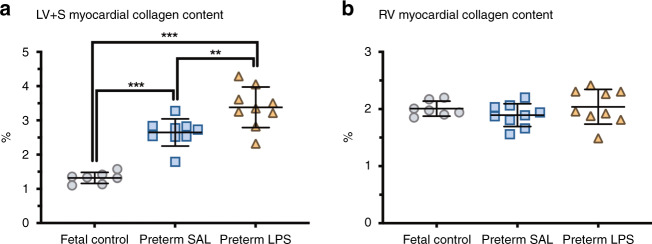
Myocardial collagen content. Levels of interstitial myocardial collagen in the left ventricle + septum (LV + S) and right ventricle (RV) of postconceptional age-matched fetal lambs (gray circles, fetal control, *n* = 7) and 7-day-old preterm lambs exposed antenatally to saline (blue squares, Preterm SAL, *n* = 9) or LPS (orange triangles, preterm LPS, *n* = 9). Statistical analyses were performed using a one-way ANOVA followed by a Bonferroni post hoc test. Data are presented as mean ± SD, ** denotes *p* < 0.01, *** denotes *p* < 0.001.

Levels of interstitial collagen were higher in the LV + S than the RV in all groups (Fig. 1). The hearts of all preterm lambs exhibited higher levels of interstitial collagen within the LV + S than fetal controls (*p* < 0.0001). Preterm lambs exposed antenatally to LPS had the highest levels of interstitial collagen deposition in the LV + S myocardium (*p* = 0.005). Collagen content within the RV was similar between groups.

### Immune cell infiltration

**Fig. 2:**
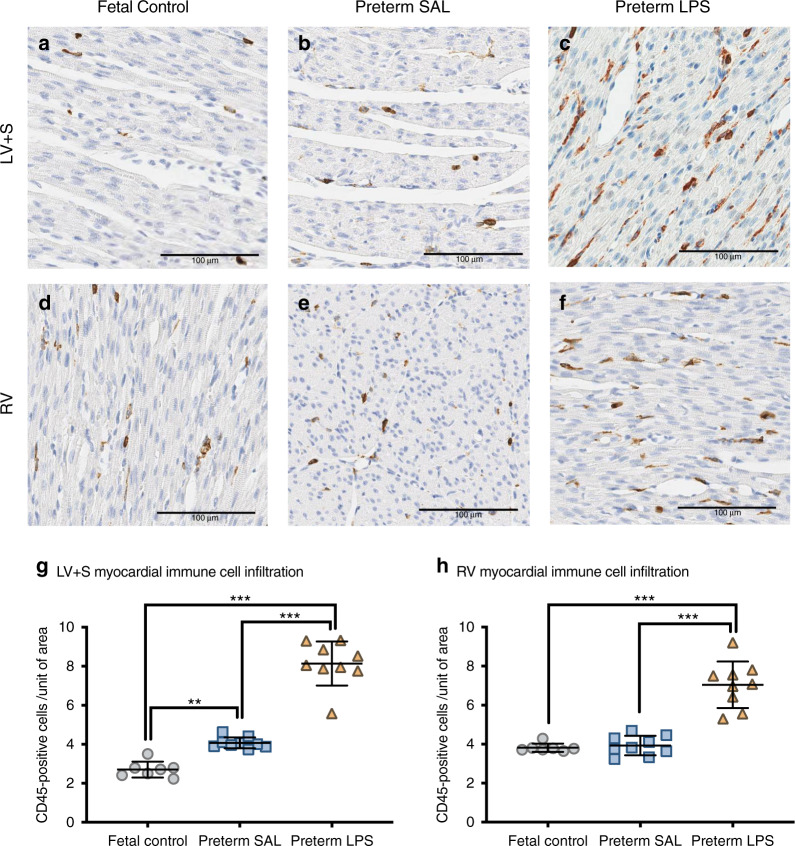
CD45^+^ immunostaining for myocardial immune cell infiltration. Representative images of immunohistochemical labeled CD45^+^ cells in the left ventricle + septum (LV + S; **a**–**c**) and right ventricle (RV; **d**–**f**) from postconceptional aged-matched fetal lambs (fetal control, *n* = 7) and preterm lambs exposed antenatally to saline (preterm SAL, n = 9) or LPS (preterm LPS, *n* = 9). Scale bar = 100 μm. Graphs show immune cell infiltration of the LV + S (**g**) and RV (**h**) in each experimental group (gray circles = fetal control; blue squares = preterm SAL; orange triangles = preterm LPS). Statistical analyses were performed using a one-way ANOVA followed by Bonferroni post hoc test. Data are presented as mean ± SD, ** denotes *p* < 0.01, *** denotes *p* < 0.001.

Overall, the number of CD45^+^ cells present in the LV + S was higher in the preterm lambs (both experimental groups) compared to the fetal controls (Fig. 2). Prior exposure to intra-amniotic LPS increased the number of CD45^+^ cells present within the LV + S of preterm lambs (*p* < 0.0001) when compared to the preterm SAL group. In the RV, the number of CD45^+^ cells present was similar between the preterm SAL group and the fetal controls. However, preterm lambs exposed to intra-amniotic LPS had a higher number of CD45^+^ cells in the RV myocardium (*p* < 0.0001).

### Cardiomyocyte proliferation

**Fig. 3:**
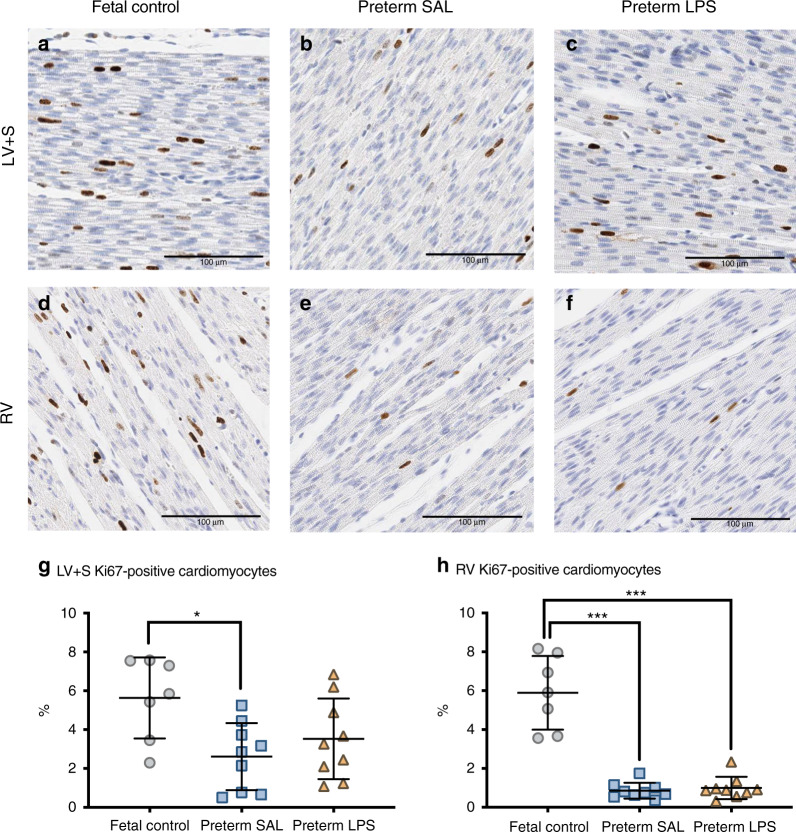
Ki67 immunostaining of proliferating cardiomyocytes. Representative images of Ki67 immunohistochemical staining in the left ventricle + septum (LV + S; **a**–**c**) and right ventricle (RV; **d**–**f**) from aged-matched fetal lambs (fetal control, *n* = 7) and preterm lambs exposed antenatally to saline (preterm SAL, *n* = 9) or LPS (preterm LPS, *n* = 9). Scale bar = 100 μm. Graphs show the proportion of Ki67 positively-labeled cardiomyocytes in the LV + S (**g**) and RV (**h**) of each experimental group (gray circles = fetal control; blue squares = preterm SAL; orange triangles = preterm LPS). Statistical analyses were performed using a one-way ANOVA followed by a Bonferroni post hoc test. Data are presented as mean ± SD, * denotes *p* < 0.05, *** denotes *p* < 0.001.

Cardiomyocyte cell cycle activity (determined by Ki67 immunostaining) in all preterm lambs was greater in the LV + S compared to the RV (Fig. 3). Preterm SAL lambs exhibited a lower proportion of Ki67-positive cardiomyocytes in the LV + S compared to fetal controls (*p* = 0.017); however, the proportion of Ki67-positive LV + S cardiomyocytes was not different between the preterm groups. In the RV, the proportion of Ki67-positive cardiomyocytes was lower in both the preterm SAL and preterm LPS groups compared to the fetal controls (*p* < 0.0001).

### Cardiomyocyte number

**Fig. 4:**
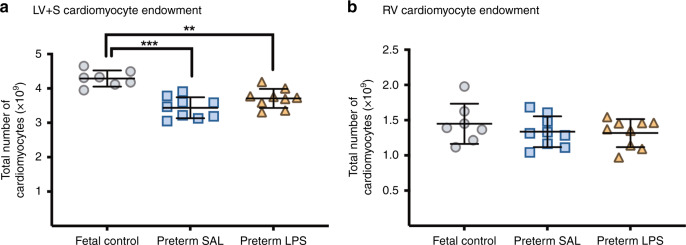
Cardiomyocyte endowment. Total number of cardiomyocytes in the left ventricle + septum (LV + S; **a**) and right ventricle (RV; **b**) of postconceptional age-matched fetal lambs (gray circles, fetal control, *n* = 7) and 7-day-old preterm lambs exposed antenatally to saline (blue squares, preterm SAL, *n* = 9) or LPS (orange triangles, preterm LPS, *n* = 9). Statistical analyses were performed using a one-way ANOVA followed by a Bonferroni post hoc test. Data are presented as mean ± SD, ** denotes *p* < 0.01, *** denotes *p* < 0.001.

The number of cardiomyocytes within the LV + S of preterm lambs was significantly reduced compared to postconceptional age-matched fetal controls (preterm SAL vs fetal control, *p* < 0.0001; preterm LPS vs Fetal Control, *p* = 0.001; Fig. 4). Cardiomyocyte endowment within the RV did not differ between groups.

### Cardiomyocyte cross-sectional area

LV + S cardiomyocyte cross-sectional area was greater in the preterm SAL (*p* = 0.008) and preterm LPS (*p* < 0.0001) lambs compared to the fetal control lambs (Table 3). In contrast, the cross-sectional area of RV cardiomyocytes was smaller in the preterm SAL (*p* = 0.03) and preterm LPS (*p* = 0.02) lambs compared to the fetal controls.

**Table 3:** Cardiomyocyte size and nuclearity.

	Fetal control (*n* = 7)	Preterm SAL (*n* = 9)	Preterm LPS (*n* = 9)
LV + S			
Cross-sectional area (µm^2^)	39.6 ± 2.2	49.5 ± 4.1*	56.5 ± 8.5*
Mononucleated (%)	44.9 ± 1.5	31.7 ± 2.4*	31.8 ± 1.3*
Binucleated (%)	55.0 ± 1.5	68.2 ± 2.4*	68.0 ± 1.5*
Multinucleated (>2 nuclei) (%)	0.1 ± 0.1	0.1 ± 0.1	0.2 ± 0.4
RV			
Cross-sectional area (µm^2^)	61.0 ± 3.5	53.3 ± 6.4*	52.8 ± 5.6*
Mononucleated (%)	38.0 ± 1.9	31.0 ± 1.0*	32.0 ± 1.0*
Binucleated (%)	61.9 ± 1.9	68.4 ± 1.1*	67.7 ± 0.7*
Multinucleated (>2 nuclei) (%)	0.1 ± 0.1	0.6 ± 0.6	0.3 ± 0.5

Cardiomyocyte size (cross-sectional area) and nuclearity from the left ventricle + septum (LV + S) and right ventricle (RV). Statistical analyses were performed using a one-way ANOVA followed by a Bonferroni post hoc test. Data presented as mean ± SD. Significance accepted at *p* *<* 0.05: *significant difference compared to fetal control.

### Cardiomyocyte nuclearity

The proportion of binucleated cardiomyocytes was greatest in the preterm hearts (Table 3). In the LV + S, the percentage of binucleated cardiomyocytes was significantly higher (*p* < 0.001), and the percentage of mononucleated cardiomyocytes was lower (*p* < 0.001) in both preterm lamb groups compared to the fetal controls.

Likewise, preterm lambs had a greater proportion of binucleated cardiomyocytes (*p* < 0.001) and fewer mononucleated cardiomyocytes (*p* < 0.0001) in the RV compared to fetal controls. In both the LV + S and RV, there were no differences in the relative proportions of mononucleated or binucleated cardiomyocytes between the preterm groups. A small number of multinucleated cardiomyocytes (>2 nuclei) were observed in both ventricles but there were no significant differences between groups.

### Gene expression in the left ventricle

**Fig. 5:**
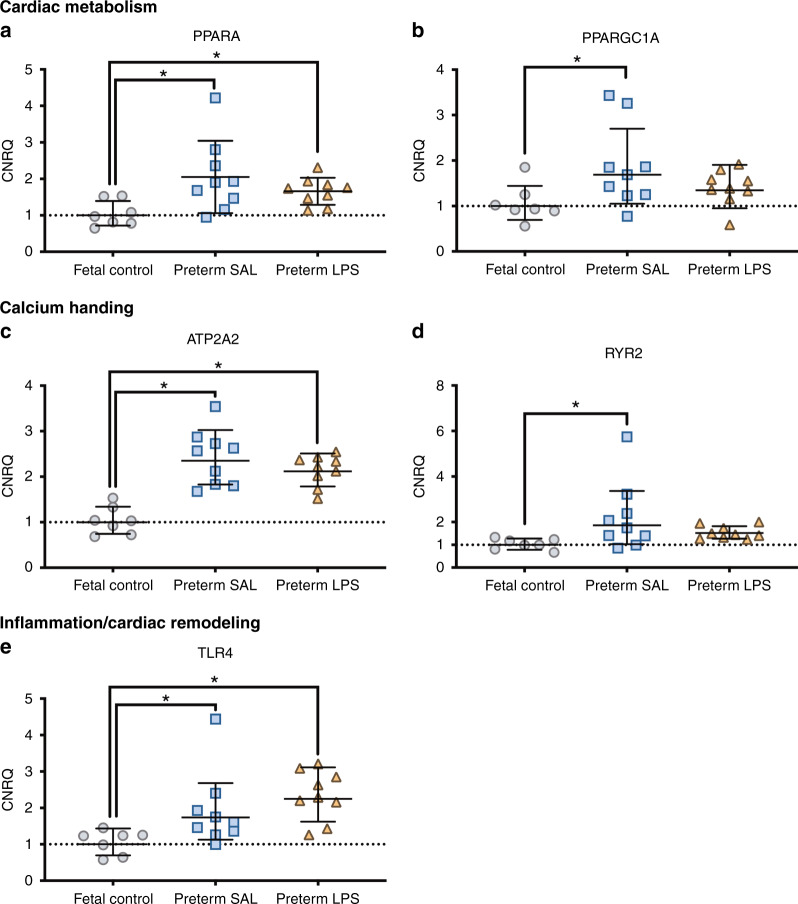
Changes in mRNA expression within the left ventricular myocardium. Genes involved in cardiac metabolism (**a**, **b**), calcium handling (**c**, **d**), and inflammation/ cardiac remodeling (**e**) were differentially expressed in the left ventricle + septum from aged-matched fetal lambs (gray circles, fetal control, *n* = 7) and preterm lambs exposed antenatally to saline (blue squares, preterm SAL, *n* = 9) or LPS (orange triangles, preterm LPS, *n* = 9). Expression of all genes was represented as the calibrated normalized relative quantity (CNRQ); normalized to YWHAZ and expressed relative to the fetal control group normalized at 1.00. Statistical analyses were performed using a one-way ANOVA followed by Tukey–Kramer post hoc test. Data are presented as mean ± SD, * denotes *p* < 0.05.

Preterm LPS lambs exhibited similar relative mRNA levels for all genes examined in the LV when compared to preterm SAL lambs. The relative mRNA levels for genes involved in cardiac metabolism (*PPARGC1A* and *PPARA*), calcium handling (*RYR2* and *ATP2A2*), and inflammation (*TLR4*) were significantly upregulated in the LV of preterm SAL lambs compared to fetal controls (*p* < 0.05) (Fig. 5). Other genes involved in angiogenesis, collagen synthesis, extracellular matrix modulation, inflammation, calcium handling and cardiac hypertrophy, and remodeling and inflammation were not differentially expressed between the experimental groups (Supplementary Table 1). The relative mRNA levels for selected genes involved in inflammation, cardiac remodeling, and hypertrophy were highly variable within groups.

### Gene expression in the right ventricle

**Fig. 6:**
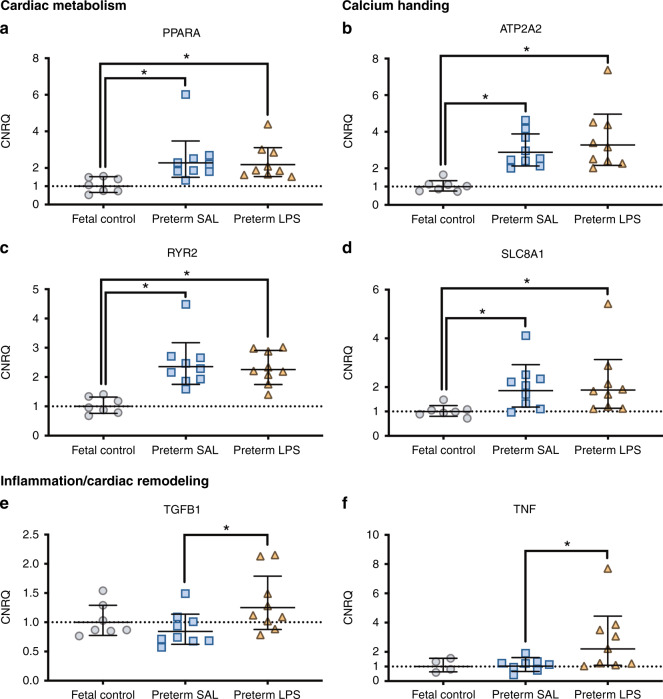
Changes in relative mRNA expression within the right ventricular myocardium. Genes involved in cardiac metabolism (**a**), calcium handling (**b**–**d**), and inflammation/ cardiac remodeling (**e**, **f**) were differentially expressed in the right ventricle from aged-matched fetal lambs (gray circles, fetal control, *n* = 7) and preterm lambs exposed antenatally to saline (blue squares, preterm SAL, *n* = 9) or LPS (orange triangles, preterm LPS, *n* = 9). Expression of all genes was represented as the calibrated normalized relative quantity (CNRQ); normalized to YWHAZ and expressed relative to the fetal control group normalized at 1.00. Statistical analyses were performed using a one-way ANOVA followed by Tukey–Kramer post hoc test. Data are presented as mean ± SD, * denotes *p* < 0.05.

The mRNA levels for genes involved in calcium handling (*SLC8A1*, *RYR2,* and *ATP2A2*) and cardiac metabolism, (*PPARA*) were higher in the RV of both the preterm SAL (*p* < 0.05) and preterm LPS (*p* < 0.05) lambs when compared to the fetal controls (Fig. 6). Preterm LPS lambs had higher *TNF* and *TGFβ1* mRNA levels compared to preterm SAL lambs (*p* < 0.05). Other genes involved in angiogenesis, collagen synthesis, extracellular matrix modulation, calcium handling, inflammation and cardiac hypertrophy, remodeling, and metabolism were not different in their relative mRNA expression between groups and high variability existed within groups (Supplementary Table 2).

## Discussion

The findings of this preclinical sheep study show the abnormal ventricular structure and altered cardiomyocyte growth in the newborn heart following preterm birth. These effects of prematurity on the heart are exacerbated when premature birth is preceded by in utero exposure to an inflammatory environment. The findings are of concern and are of major clinical relevance, given that ~10% of all live births are preterm,^1^ and chorioamnionitis is a common antecedent of premature delivery and likely common for many term births.^3^

### Collagen deposition is increased in the preterm left ventricle and exacerbated when preceded by intrauterine inflammation

The extracellular matrix of the heart, composed predominantly of types I and III collagen, serves as a bioactive mechanical scaffold and is crucial for normal cardiac function.^35^ Increases in interstitial collagen adversely affect both the electrical conductivity and contractility of the myocardium, and are thus implicated in the pathogenesis of cardiac pathophysiology.^35–37^ It is likely that in those born preterm, the altered gross structure of the heart in adulthood, such as displacement of the apex and shorter ventricles with smaller cavities but greater mass,^19,20^ may be the repercussions of early life changes to the myocardial collagen scaffold in the LV + S. Recent data from Lewandowski et al.^21^ provide the first evidence of increased diffuse myocardial fibrosis in the LV in young adults born preterm, with the proportion of fibrosis increasing in proportion to the degree of prematurity. The changes to the LV structure and myocardial collagen scaffold associated with the degree of prematurity have direct implications on cardiac function, including impaired diastolic function.^21^ Increased LV + S collagen may also play a role in the increased vulnerability to heart failure in children born preterm.^38^ The findings in this study and in previous human and animal studies^21,22,24^ of increased myocardial collagen within the LV + S support the concept of an altered cardiac scaffold in the preterm heart. Given that the levels of myocardial collagen within the LV + S were exacerbated when the preterm lambs were exposed antenatally to LPS, this indicates that the collagen scaffolding framework can be impacted upon by both antenatal and postnatal factors. Further investigations into the increased collagen deposition in the immature preterm heart should examine the cross-linking of collagen fibrils within fibers as a determinant of the resistance of collagen fibers to degradation,^39^ and ratios of type I to type III collagen as an indicator of cardiac stiffening.^40^

### The right ventricle may be better prepared for preterm birth with less adverse consequences in the neonatal period

In contrast to the LV + S, the collagen content of the RV is not influenced by exposure to intrauterine inflammation and/or prematurity. Although there is evidence to suggest maternal undernutrition during late gestation triggers remodeling of the RV,^41^ these changes were not observed with exposure to intrauterine inflammation. The reason for the observed differences in the impact of preterm birth on LV + S and RV interstitial collagen content is unknown. It is conceivable that the extracellular framework is established earlier and cardiomyocyte maturation occurs earlier in the RV when compared to the LV; given that the RV is the dominant chamber of the prenatal heart (responsible for 66% of the fetal cardiac output).^42^ Such accelerated development of the RV would lessen the adverse impact of preterm birth on the RV myocardium. The absence of a difference in cardiomyocyte endowment within the RV between all groups in the current study supports this notion.

The possibility remains that initiation of changes to the LV architecture early in life may impact function in both ventricles and lead to postnatal changes to both LV and RV structure in later life. Notably, in contrast to our findings, other studies^22,23^ in animal models of prematurity exposed to antenatal betamethasone identified increased fibrosis in both the LV and RV; however, the analyses were conducted at later ages and therefore prematurity-associated changes to the myocardium may occur with aging. Cardiovascular magnetic resonance imaging studies in young adults born preterm demonstrate the consequences of preterm birth are greater on RV structure and function^20^ than the LV^19^ and are independent of changes in pulmonary physiology.^43^ Further investigation is required to determine how the preterm heart grows postnatally and how postnatal factors influence the long-term growth in both ventricles.

### Intrauterine inflammation leads to myocardial inflammation of the preterm heart

The current study demonstrates clearly that exposure to an inflammatory environment prior to preterm birth prompts a myocardial immune response, thus rendering greater vulnerability to preterm birth. The heart is a target for LPS-induced inflammation: previous studies show that *TLR4* expression is upregulated in cardiomyocytes and leukocytes in response to LPS, and upregulation of *TLR4* expression is linked to cardiac dysfunction.^44,45^ The expression of genes involved in cardiac morphogenesis, remodeling and vasculogenesis is disrupted rapidly following exposure of fetal non-human primates to intrauterine inflammation.^46^ In addition, exposure to an inflammatory stimulus in mid-gestation (*Candida albicans*) and late-gestation (LPS) is linked to abnormal cardiac growth and function in fetal sheep.^8,47^ In the present study, the LV myocardium of preterm lambs shows increased expression of *TLR4* and an increased presence of immune cells, which is exacerbated by antenatal exposure to an inflammatory environment. The previous findings^22^ of inflammatory cell infiltration into the LV following preterm birth suggests that preterm birth acts as an insult to the LV myocardium. In contrast, increased expression of inflammatory genes and increased immune cell presence in the RV is observed only in preterm lambs antenatally exposed to LPS. A number of key genes related to inflammation and cardiac remodeling may have been upregulated in utero and/or in the immediate period after preterm birth depending on the antenatal experiences. However, differences in gene expression may be normalized by the time of analysis at postnatal day seven.

### Altered cardiomyocyte growth kinetics as a result of preterm birth

Direct effects of prematurity on the hallmarks of cardiomyocyte maturation are observed in both ventricles. The prematurely reduced cardiomyocyte proliferation and increased proportion of binucleated cardiomyocytes associated with exposure to the postnatal environment suggest that cardiomyocytes were undergoing accelerated maturation and differentiation^48^ in comparison to age-matched fetal controls. Cardiomyocyte growth is achieved mainly via hypertrophy in postnatal life.^16^ The increase in the size of the LV + S cardiomyocytes one week after preterm delivery is consistent with accelerated cardiomyocyte maturation and growth resulting from changes in hemodynamic forces.^31^ Antenatal LPS exposure, mimicking the clinical condition of chorioamnionitis, was expected to exacerbate the observed effects of prematurity on cardiomyocyte maturation and growth because accelerated cardiomyocyte maturation was observed in fetal lambs exposed to LPS for 7 days.^8^ Our findings, to the contrary, at one week after preterm birth were unexpected, raising the possibility that the antenatal LPS effects may be overridden by the postnatal cellular response to prematurity. The respective differences in cardiomyocyte size in the LV + S and RV postnatally likely reflect the transition from fetal to postnatal circulation: changes to systemic and pulmonary blow flow and vascular resistance, as well as ventricular function and dominance.^49,50^ Indeed, hemodynamic pressures and other physiological stimuli are strong modulators of cardiomyocyte growth and function.^51–54^ From a clinical perspective, modulators of cardiomyocyte growth during the perinatal period may have long-term consequences on the preterm-born individual.

The dynamics of cardiomyocyte growth and maturation change in late gestation, where there is a decline in proliferative growth and concomitant maturation, differentiation, binucleation, and hypertrophic growth of the cardiomyocytes.^31,48^ In our model, preterm birth occurs during this crucial developmental window. Therefore, late gestational exposure to chorioamnionitis and/or premature exposure to the extrauterine environment may perturb cardiomyocyte maturation and endowment. It is possible that the synthetic rise in corticosteroids due to the maternal administration of betamethasone may also accelerate fetal cardiomyocyte growth and maturation in preparation for postnatal life and hence influenced the number of cardiomyocytes.^55,56^ Importantly, the number of cardiomyocytes within the LV + S reduced consequent to prematurity, which is likely attributed to smaller body size. Given the limited replicative capacity of cardiomyocytes postnatally, the reduced endowment of LV + S cardiomyocytes following preterm birth may adversely impact lifelong functional reserve and the adaptive (structural and functional) capabilities of the myocardium for physiological hypertrophy in those individuals born preterm. Recent studies highlight impaired LV^57,58^ and RV^59,60^ function of preterm-born young adolescents and adults when physiologically challenged with exercise, which is suggestive of reduced cardiac functional reserve. Similarly, a number of clinical and experimental reports highlight a unique cardiac phenotype in those born preterm compared to term counterparts, including altered cardiac function, evidence of myocardial remodeling, and changes to cardiomyocyte maturation (including nuclearity, size, and ploidy).^17–20,22,23,61^

### Limitations

The preterm lambs in this study were treated pragmatically according to their individual needs (similar to preterm infants), which may contribute to some of the variability within the experimental groups. The findings of the preterm saline group could be a consequence of antenatal betamethasone and/or the consequences of postnatal treatment because the naïve fetal controls were not exposed to antenatal medroxyprogesterone or betamethasone. Prior experience of administering ewes betamethasone at ~126 days of gestation resulted in preterm delivery after 48 h therefore a fetal control group with the same antenatal experiences as the preterm lambs were omitted at the time of the study. Given that the fetal controls were not exposed to the same extrauterine hemodynamic and physiological conditions, it is appropriate to consider them as a control of normal in utero vs ex utero development, rather than as a direct control against the preterm groups. Finally, the study was designed to assess short-term outcomes, therefore, as the cardiovascular disease takes years to manifest it is of importance for future studies to assess the long-term cardiac effects.

## Conclusion

In conclusion, preterm birth has an adverse impact on cardiac structure and cardiomyocyte growth kinetics in the neonatal heart within the first week of postnatal life. The further complication of intrauterine inflammation exposure preceding preterm birth prompts a myocardial immune response and exacerbates remodeling of the immature heart. Further research into the effects of an inflammatory intrauterine environment on cardiac development is required, given that chorioamnionitis is a major contributor to preterm birth. The findings of this study, combined with other recent reports, highlight the need to monitor cardiovascular health throughout life in individuals born preterm, especially those that were exposed to chorioamnionitis, as the lifelong consequences to cardiovascular health are of major clinical importance.

## Supplementary information

Supplementary Material
