# Supplementary material for: Intrauterine inflammation exacerbates maladaptive remodeling of the immature myocardium after preterm birth in lambs
Source: Pediatr Res. 2022 Mar 11;92(6):1555–65. doi: 10.1038/s41390-022-01955-7 (PMC9771797; doi:10.1038/s41390-022-01955-7)
Supplement: Supplementary file 1 — Supplementary Material [file 41390_2022_1955_MOESM1_ESM.pdf]

Supplementary Material

Supplementary Methods

Immunohistochemical analysis of cellular proliferation and Immune cell infiltration

Immunohistochemical detection of cellular proliferation (via a Ki67 antibody), and immune cell infiltration (via a CD45 (common leukocyte antigen) antibody) was undertaken in paraffin-embedded ventricular sections using a DAKO Austostainer Plus staining system.[1] The deparaffinised heart sections initially underwent antigen retrieval in sodium citrate buffer followed by blocking with real peroxidase block and DAKO serum-free protein block before staining. The sections were then incubated with either the Ki67 antibody (1:100; mouse monoclonal M7240, Dako, Denmark) or the CD45 antibody (1:500; mouse monoclonal MCA2220GA, BioRad). Histological sections of lamb ileum were used as a positive and a negative control for Ki67 AND CD45 immunohistochemical staining. Following a series of buffer washes, the sections were incubated with an anti-mouse Dako EnVision + System HRP Labeled Polymer secondary antibody (ready to use; K4001, Dako) and positive staining visualised by incubation in 3, 3′-diaminobenzidine in chromogen solution (DAB; K3468, Invitrogen). The slides immunohistochemically stained for Ki67 or CD45 were scanned at 40x magnification and exported into Aperio Imagescope for the quantification of cellular proliferation or immune cell infiltration, respectively.

Quantification of cardiomyocyte cross-sectional area and nuclearity

Cardiomyocyte cross-sectional area and nuclearity was quantified in 5 µm paraffin sections stained with a cell membrane stain, Wheat Germ Agglutinin (WGA) conjugated to Alexa-Fluor 488 (Invitrogen, USA), and a DNA stain to identify the cell nucleus, DAPI (4', 6-diamidino-2-phenylindole; Invitrogen).[2,3] The deparaffinised slides were incubated with the stain (10 µg/mL WGA-AF488 and 1 µg/mL DAPI in PBS) at room temperature for 25 minutes in a dark chamber protected from light exposure and then washed twice in PBS and coverslipped with glass coverslips using ProLong® Gold antifade reagent (Invitrogen).

Images were acquired on a Nikon C1 confocal microscope (Tokyo, Japan) at Monash Micro Imaging. Eight random fields in cross section and another eight random fields in longitudinal section were selected per slide and imaged in a Z-stack for cross sectional area and nuclearity analyses. Captured images were opened in Fiji (Version 2.0.0) where the cross-sectional area was determined by using a calibrated area tool to draw an outline around a cardiomyocyte. Cardiomyocytes were only measured when it was in true cross-section with a centrally located nucleus.[2] The cross-sectional area of more than 300 cardiomyocytes was measured per ventricle. Images in the longitudinal plane were analysed through the Z-stack to visualise and quantify nuclearity in more than 250 cardiomyocytes.[4]

Stereological estimation of cardiomyocyte endowment

Glycolmethacrylate-embedded tissue blocks were sectioned exhaustively at a thickness of 20 µm, with every 40th section used for counting cardiomyocyte nuclei using an optical disector. Sections were stained with Harris's Haematoxylin (Amber Scientific, Queensland, Australia) for identification of cardiomyocyte nuclei.[5,6] The optical disector-fractionator technique[7] was employed to estimate cardiomyocyte number.[4,5,8] Proprietary software (C.A.S.T, Computer Aided Stereological Toolbox; CAST 2002, Olympus, Alberstlund, Denmark) was used to count cardiomyocyte nuclei observed at a magnification of 100x on an Olympus BX4 microscope (Olympus, Tokyo, Japan); the microscope had a motorised stage and self-calibrating Z axis sensor (Heidenheim, Germany). C.A.S.T generation of uniform systematic sampled fields (2000 µm step length) were superimposed with an unbiased counting frame (538.4 µm^2). The number of cardiomyocyte nuclei were then counted within the middle 10 µm of the section using an optical disector approach. The total number of cardiomyocyte nuclei per ventricle were subsequently calculated by multiplying the number of nuclei counted using the optical disector with the reciprocal of the sampling fractions. Total cardiomyocyte number was then determined by adjusting for cardiomyocyte nuclearity (number of nuclei per cardiomyocyte).[4,5]

RNA extraction

The RV and LV were analysed separately for molecular analyses. Snap frozen ventricular tissue was lysed with TRIzol® Reagent (Ambion, Life Technologies, Carlsbad, USA) and homogenised (T 10 basic ULTRA-TURRAX®; IKA®-Werke, Staufen, Germany). Chloroform (VWR International, Radnor, USA) was added to the tissue homogenate and centrifuged at 12000 x g for 15 minutes. The supernatant was transferred into an RNase-free tube and mixed vigorously with an equal volume of 70 % ethanol. The supernatant/ethanol mixture was transferred onto the spin cartridge within a collection tube (PureLink™ RNA Mini Kit; Invitrogen, Life Technologies). The collection tubes were centrifuged at 12000 x g for 15 seconds to bind RNA to the spin cartridge membrane and the flow through discarded. The spin cartridge was washed with Wash Buffer I, then centrifuged and the flow-through discarded. PureLink® DNase mixture was directly added onto the surface of the spin cartridge membrane and incubated for 15 minutes. The spin cartridge membrane underwent a series of washes, using Wash Buffer I and Wash Buffer II, followed by centrifugation at 12000 x g for 15 seconds. The collection tube was discarded and the spin cartridge inserted into a recovery tube. RNase-free water was added to the membrane to elute RNA. After incubation, the recovery tube was centrifuged for 2 minutes at room temperature. The purified RNA was quality checked for RNA yield using spectrophotometry (NanoPhotometer® N50; Implen, Munchen, Germany). Total RNA was stored at -80 °C.

cDNA Synthesis

Complimentary DNA (cDNA) was synthesised from total RNA using the SuperScript® III First-Strand Synthesis System (Invitrogen, Life Technologies). RNA was diluted with RNase free water and aliquoted onto a plate. The RNA was primed with 10 mM dNTP mix and random hexamers and denatured at 65 °C for 5 minutes and put on ice for 1 minute. The cDNA synthesis mix (prepared with: 5 X First Strand buffer, 0.1 M DTT, RNaseOUT™ and SuperScript® III Reverse Transcriptase) was added to the RNA/primer mixture and centrifuged at 500 x g for 1 minute. A negative control, –Reverse Transcriptase (-RT)

control, was prepared with all components for cDNA synthesis except for the SuperScript® III Reverse Transcriptase to determine the presence of genomic DNA contamination in the cDNA sample. The plate was incubated in the thermal cycler (Veriti™ 96-Well Thermal Cycler; Applied Biosystems, Life Technologies) to undergo annealing (25 °C for 5 minutes), cDNA synthesis (50 °C for 60 minutes), termination of reaction (70 °C for 15 minutes) and cooling (4 °C for 10 minutes). The cDNA was stored at -20 °C.

Gene Expression

All samples, cDNA and –RT control samples, were quality checked by real-time PCR, using predesigned sheep *GAPDH* primers in combination with SYBR™ chemistry run on the 7900HT Fast Real-Time PCR System (Applied Biosystems, Life Technologies). Pre-amplification was conducted on the samples to increase the number of copies of each gene to levels detectable, according to the Fluidigm® gene expression specific target amplification reference protocol. The TaqMan® assays (Applied Biosystems, Life Technologies,) were provided as 20X forward and reverse primer and probe mixes; each primer was at a concentration of 18 µM and each probe was at a concentration of 4 µM. TaqMan® assays were firstly pooled by combining 4 µL of each of the 20X Taqman assays and 296 µL Tris EDTA buffer for a final volume of 300 µL. The final concentration of each assay was 0.2X (180 nM). Preamplification allowed multiplex amplification: 3.75 µL of Sample Pre Mix (TaqMan® PreAmp Master Mix and Pooled Taqman assays; Applied Biosystems, Life Technologies) was combined with 1.25 µL cDNA or -RT control samples for a final reaction volume of 5 µL per sample. A no template control (NTC) was also included and all samples were preamplified for 14 cycles. Following preamplification, reaction products were diluted 1:5 by adding 20 µL Tris EDTA buffer to the final 5 µL reaction volume for a total volume of 25 µL.

Assays and samples were combined on a Biomark™ HD (Fluidigm, South San Francisco, USA) gene expression integrated fluidic circuit, as per the Fluidigm® real-time PCR protocol.

Briefly, 3 μL of each assay at a final concentration of 10x was added to each assay inlet port and 3 μL of diluted sample to each sample inlet port according to the Chip Pipetting Map; selected samples were run in duplicate. The real-time PCR was conducted on the 7900HT Fast Real-Time PCR System (Applied Biosystems, Life Technologies) and the reaction commenced at 50 °C for 2 minutes, then 95 °C for 10 minutes, and 40 cycles of 95 °C for 15 seconds followed by 60 °C for 60 seconds. The data were analysed with Fluidigm Real-Time PCR analysis software (version 4.1.1; Fluidigm, South San Francisco, USA) for the generation of Ct values for each sample.

Supplemental Tables

Supplemental Table 1: Relative expression of genes in the left ventricular myocardium

Gene of interest	Fetal Control	Preterm SAL	Preterm LPS
COL1A1	1.00 ± 0.33	0.94 ± 0.37	0.65 ± 0.34
COL3A1	1.00 ± 0.35	0.67 ± 0.39	0.53 ± 0.16
GATA4	1.00 ± 0.43	1.46 ± 0.59	1.43 ± 0.37
IGF-1	1.00 ± 0.19	1.69 ± 0.70	2.06 ± 2.11 *****
IL-1b	1.00 ± 0.85	1.18 ± 4.53	2.98 ± 12.63
IL-18	1.00 ± 1.19	1.00 ± 0.97	1.51 ± 0.92
MMP9	1.00 ± 1.47	1.27 ± 5.55	1.77 ± 2.12
MYH7	1.00 ± 0.34	1.23 ± 1.27	0.90 ± 0.35
NCX1	1.00 ± 0.30	1.70 ± 1.34	1.53 ± 0.43
NPPA	1.00 ± 1.59	1.51 ± 3.89	1.18 ± 1.31
TIMP2	1.00 ± 0.33	1.26 ± 0.48	1.06 ± 0.37
TNF	1.00 ± 0.63	2.64 ± 1.29	2.59 ± 4.70
TGFβ1	1.00 ± 0.29	1.04 ± 0.28	0.94 ± 0.32
VEGFA	1.00 ± 0.66	1.00 ± 0.66	0.92 ± 0.27

*Expression of all genes in the left ventricle from aged-matched fetal lambs (Fetal Control, n=7) and preterm lambs exposed antenatally to saline (Preterm SAL, n=9) or LPS (Preterm LPS, n=9). Gene expression was represented as the calibrated normalised relative quantity (CNRQ); normalised to YWHAZ and expressed relative to the Fetal Control group normalised at 1.00. Data are presented as mean ± SD, * denotes significant difference compared to Fetal Control.*

Supplemental Table 2: Relative expression of genes in the right ventricular myocardium

**Gene of interest**	**Fetal Control**	**Preterm SAL**	**Preterm LPS**
COL1A1	1.00 ± 0.43	0.75 ± 0.44	1.30 ± 1.12
COL3A1	1.00 ± 0.26	0.96 ± 0.44	1.13 ± 0.52
GATA4	1.00 ± 0.24	1.18 ± 0.59	1.78 ± 0.93 *
IGF-1	1.00 ± 0.39	1.50 ± 0.65	2.11 ± 1.86 *
IL-1b	1.00 ± 1.03	1.00 ± 2.08	2.99 ± 27.22
IL-18	1.00 ± 0.64	0.94 ± 0.53	0.67 ± 0.53
MMP9	1.00 ± 0.80	2.96 ± 5.92	3.00 ± 16.76
MYH7	1.00 ± 0.22	0.86 ± 0.54	0.91 ± 0.49
NPPA	1.00 ± 1.04	0.40 ± 1.51	0.40 ± 0.66
PPARGC1A	1.00 ± 0.55	1.10 ± 0.34	1.16 ± 0.37
TIMP2	1.00 ± 0.29	1.12 ± 0.61	1.39 ± 0.96
TLR4	1.00 ± 0.66	1.42 ± 0.72	1.91 ± 0.90 *
VEGFA	1.00 ± 0.88	0.75 ± 0.34	1.10 ± 0.42

*Expression of all genes in the right ventricle from aged-matched fetal lambs (Fetal Control, n=7) and preterm lambs exposed antenatally to saline (Preterm SAL, n=9) or LPS (Preterm LPS, n=9). Gene expression was represented as the calibrated normalised relative quantity (CNRQ); normalised to YWHAZ and expressed relative to the Fetal Control group normalised at 1.00. Data are presented as mean ± SD, * denotes significant difference compared to Fetal Control.*

References

1 Flores, T. J. et al. Morphology and Function of the Lamb Ileum Following Preterm Birth. *Front Pediatr* **6**, 8 (2018).

2 Goh, J. M. et al. Alcohol Exposure During Late Gestation Adversely Affects Myocardial Development with Implications for Postnatal Cardiac Function. *American journal of physiology Heart and circulatory physiology* **300**, H645-651 (2011).

3 Bensley, J. G., De Matteo, R., Harding, R. & Black, M. J. Three-Dimensional Direct Measurement of Cardiomyocyte Volume, Nuclearity, and Ploidy in Thick Histological Sections. *Sci Rep* **6**, 23756 (2016).

4 Bensley, J. G., Stacy, V. K., De Matteo, R., Harding, R. & Black, M. J. Cardiac Remodelling as a Result of Pre-Term Birth: Implications for Future Cardiovascular Disease. *Eur Heart J* **31**, 2058-2066 (2010).

5 Corstius, H. B. et al. Effect of Intrauterine Growth Restriction on the Number of Cardiomyocytes in Rat Hearts. *Pediatr Res* **57**, 796-800 (2005).

6 Lim, K. J., Zimanyi, M. A. & Black, M. J. Effect of Maternal Protein Restriction in Rats on Cardiac Fibrosis and Capillarization in Adulthood. *Pediatr Res* **60**, 83-87 (2006).

7 Bruel, A. & Nyengaard, J. R. Design-Based Stereological Estimation of the Total Number of Cardiac Myocytes in Histological Sections. *Basic Res Cardiol* **100**, 311-319 (2005).

8 Stacy, V. et al. The Influence of Naturally Occurring Differences in Birthweight on Ventricular Cardiomyocyte Number in Sheep. *Anat Rec (Hoboken)* **292**, 29-37 (2009).
